# Dietary Intake, Nutritional Adequacy, and Food Sources of Selected Antioxidant Minerals and Vitamins; and Their Relationship with Personal and Family Factors in Spanish Children Aged 1 to <10 Years: Results from the EsNuPI Study

**DOI:** 10.3390/nu14194132

**Published:** 2022-10-05

**Authors:** Casandra Madrigal, María José Soto-Méndez, Ángela Hernández-Ruiz, María Dolores Ruiz-López, María de Lourdes Samaniego-Vaesken, Teresa Partearroyo, Gregorio Varela-Moreiras, Ángel Gil

**Affiliations:** 1 Department of Nutrition and Food Science, Faculty of Pharmacy, University of Granada, 18071 Granada, Spain; 2 Iberoamerican Nutrition Foundation (FINUT), 18016 Granada, Spain; 3 Biomedical Research Center, Institute of Nutrition and Food Technology “José Mataix”, University of Granada, 18011 Granada, Spain; 4 Grupo USP-CEU de Excelencia “Nutrición Para la Vida (Nutrition for Life)”, Ref: E02/0720, Departamento de Ciencias Farmacéuticas y de la Salud, Facultad de Farmacia, Universidad San Pablo-CEU, CEU Universities, 28660 Boadilla del Monte, Spain; 5 Physiopathology of Obesity and Nutrition (CIBEROBN), Instituto de Salud Carlos III (ISCIII), 28029 Madrid, Spain; 6 Department of Biochemistry and Molecular Biology II, University of Granada, 18071 Granada, Spain

**Keywords:** antioxidants, vitamins, minerals, infant formula, food sources, dairy products, Spanish children, EsNuPI study

## Abstract

Minerals and vitamins involved in the antioxidant defense system are essential for healthy growth and proper development during infancy. Milk and dairy products are of particular importance for improving the supply of these nutrients to children. Indeed, the present study aimed to evaluate the nutrient intake and food sources of zinc (Zn), selenium (Se), retinol and carotenoids (sources of vitamin A), and vitamins C and E, and to analyze their relationships with personal and familiar factors in Spanish children from the EsNuPI study. One subpopulation representative of the Spanish population from 1 to <10 years old (*n* = 707) (reference group, REF) who reported consuming all types of milk over the last year, and another subpopulation of the same age who reported consuming fortified milk formulas (FMFs) (including follow-on formula, young child formula, growing up milk, toddler’s milk, and enriched and fortified milk) (*n* = 741) (fortified milk consumers, FMCs) completed two 24 h dietary recalls used to estimate their nutrient intakes and to compare them to the European Food Safety Authority (EFSA) Dietary Reference Values (DRVs). The REF reported higher median intakes than FMCs for Se (61 µg/kg vs. 51 µg/kg) and carotenoids (1079 µg/day vs. 998 µg/day). Oppositely, FMCs reported higher intakes than REF for Zn (7.9 mg/day vs. 6.9 mg/day), vitamin A (636 µg/day vs. 481 µg/day), vitamin E (8.9 mg/day vs. 4.5 mg/day), vitamin C (113 mg/day vs. 71 mg/day), and retinol (376 µg/day vs. 233 µg/day). In the REF group, more than 50% of the children met the EFSA recommendations for Zn (79.6%), Se (87.1%), vitamin A (71.3%), and vitamin C (96.7%), respectively. On the other hand, 92.2% were below the EFSA recommendations for vitamin E. In the FMC group, more than 50% of the children met the EFSA recommendations for Zn (55.2%), Se (90.8%), vitamin A (75.7%), vitamin E (66.7%), and vitamin C (100%). We found statistically significant differences between subpopulations for all cases except for Se. In both subpopulations, the main sources of all antioxidant nutrients were milk and dairy products. For carotenoids, the main sources were vegetables and fruits followed by milk and dairy products. A high percentage of children had vitamins A and E intakes below the recommendations, information of great importance to stakeholders. More studies using intakes and biomarkers are needed, however, to determine an association with diverse factors of oxidative damage.

## 1. Introduction

Healthy eating habits contribute to adequate nutrition and a healthful childhood, which could have an impact not only in the short term but also in adulthood [1,2]. Children with inadequate dietary intakes in infancy are susceptible to developing early overweight and obesity, as well as other associated chronic diseases during adulthood and even during childhood [3,4]. 

Worldwide, childhood obesity is associated with a subclinical inflammatory condition and oxidative stress (OS) that could be implicated in the development of chronic diseases [5,6,7]. OS can be defined as alterations in the pro/antioxidant balance that are cell-damaging due to the excessive generation of strongly reactive oxygen and nitrogen species [8,9,10,11]. OS leads to the oxidation of biomolecules with the consequent loss of their biological functions, as well as to homeostatic imbalance and oxidative damage to cells and tissues [11,12,13,14]. The chronicity of OS has important implications for the development of non-communicable chronic diseases including obesity, atherogenesis, diabetes, and cancer [8,15,16,17].

Scientific evidence suggests that several micronutrients, including many antioxidant minerals and vitamins such as selenium (Se), zinc (Zn), vitamins A, C, and E are involved in pathways that can modulate the OS and the function of either inactivating reactive oxygen species and/or free radicals’ properties or, in turn, to work in conjunction with enzymatic antioxidation reactions, consequently protecting against oxidative damage [8,12,18,19]. An inadequate intake of these nutrients might promote the proliferation of reactive oxygen species, which are involved/play a key role in several disease processes, including chronic diseases [20,21,22,23].

Generally, changes in dietary patterns lead to changes in nutrient intakes; therefore, it is important to know the status of antioxidant vitamins in the Spanish infant population. In the “Alimentando la Salud del Mañana” (ALSALMA) (*n* = 123) study, the percentage of children below the Recommended Dietary Allowances (RDAs)/Adequate Intakes (AIs) for vitamins A and E was high and increased with age. For instance, for vitamin A, the percentage of children below recommendations increased from 23% in children aged 1 to 2 years to 35% in children aged 2 to 3 years, while for vitamin E, the percentage increased from 40% to 53% [24].

The National Dietary Survey on Child and Adolescent Population project in Spain (ENALIA) (*n* = 1295) reported that the percentage of children below the Estimated Average Requirement (EAR) for Zn, Se, vitamin A, and vitamin C ranged from 0 to 3%. However, for vitamin E, the percentage below EAR was 22% for children aged 1 to 13 years; this percentage increased with age [25]. According to the Anthropometry, Intake, and Energy Balance Study (ANIBES), 31% of children aged 9 to 12 years (*n* = 213) do not meet the EFSA-recommended intakes for zinc (31%); vitamin A (36%); vitamin E (66%); vitamin C (37%); and Se (4%) [26].

Internationally, the National Health and Nutrition Examination Survey 2011–2016 (NHANES) (*n* = 9848) concluded that 69% of children in the United States of America (USA) aged 1–6 years had intakes below the EAR for vitamin E, whereas only 1% were below the EAR for vitamin A, vitamin C, and Zn [27]. In a cross-national comparison of four countries (Germany, Russia, USA, and Brazil) inadequate intakes of >20% were observed for vitamins E and A [28].

In Europe, deficiencies in micronutrients are especially related to the quality of the diet but not to the quantity of food consumed. For this reason, the risks of micronutrient deficiencies may persist in developed countries that are resource-rich areas [29,30,31]. Although needed in small amounts, micronutrients are essential for the healthy development, maintenance, and function of cellular processes and general well-being. Therefore, fortified milk formulas (FMFs) or growing up/toddler formulas have been designed as an alternative to cow’s milk (CM), as these types of milk are enriched and fortified with nutrients that are often in short supply, such as antioxidant minerals and vitamins, and although it is not necessary for adequate nutrition, FMFs can prevent the development of nutritional deficits or excesses during this period [32,33].

For all these reasons, the “Nutritional Study in the Spanish Pediatric Population” (EsNuPI) study selected two subpopulations, one reference group (REF) and one fortified milk consumers (FMCs). The REF group consumed all types of milk, and FMCs drank FMF regularly (including fortified milk, young child formula, enriched milk, follow-on formula, growing-up milk, and toddler’s milk). 

The objectives of the present study were: (1) to estimate the intake of antioxidant minerals and vitamins in the EsNuPI REF and FMC subpopulations divided into three specific age groups and to compare these data with the Dietary Reference Values (DRVs) published by EFSA [34]; (2) to investigate whether the consumption of dairy products (including CM or FMFs) is associated with nutrient adequacy of antioxidant minerals and vitamins in children; (3) to assess the influences of different sociodemographic aspects on antioxidant intake; (4) to describe the main food sources providing antioxidant minerals and vitamins. 

## 2. Materials and Methods

### 2.1. Study Design and Methods

The EsNuPI study is a prospective, observational, cross-sectional study, developed from the last quarter of 2018 to January 2019. Its design, protocol, and methodology has been previously published [35]. The fieldwork was performed amongst Spanish children living in urban areas with >50,000 inhabitants, distributed according to Nielsen Spanish areas (9). Two subpopulations were selected for the EsNuPI study: one representative of the urban Spanish children (REF) from 1 to <10 years old consuming all types of milk in the last 12, and one entitled fortified milk consumers (FMCs) from the same age group who had consumed FMF in the last 12 months. The term FMF included: infant formula, follow-on milk formula, toddler’s milk formula (also termed young child formula and, in Spain, “growing up” milk formula), and fortified milk formula (e. g., docosahexaenoic acid (DHA), calcium, iron, vitamin D, vitamin E, etc.).

For the final sample, 1514 parents or caregivers signed consent forms and completed the first interview (face-to-face) providing sociodemographic information, a quantitative food frequency questionnaire (FFQ), a physical activity and sedentary behavior questionnaire (PABQ), and the first 24 h dietary recall (24 h DR).

For the present article, we used data from the 1448 children who completed the two 24 h DR. The REF group represented 48.8%; being a reference to the Spanish population implies that some of the children reported consuming FMF (*n* = 49/7%) and some of them (*n* = 17/2%) reported not consuming milk in the 24 h DR. The FMC group represented 51.1%. The subpopulations were stratified by three age groups: 1 to <3 years old (31.5%); 3 to <6 years old (34.9%); and 6 to <10 years old (33.6%).

The Ethical Committee of the University of Granada approved the study protocol (No. 659/CEIH/2018) and then registered it on ClinicalTrials.gov (Unique Protocol ID: FF01/2019). The ethical principles for medical research involving human subjects included in the declaration of Helsinki were followed to develop this study.

### 2.2. Dietary Assessment and Data Processing

Parents or caregivers were used as a proxy to determine children’s dietary intake; therefore, they completed one face-to-face and one telephone 24 h DR on non-consecutive days, including weekdays and weekend days. These dietary recalls were carried out in the company of the children when they were older to facilitate the obtainment of this information and to try to minimize the biases inherent to this type of tool. It is a common practice in Spain that parents receive the menu offered to the children monthly and, for pre-schoolers and young school children, they receive an additional daily report about possible menu changes and the consumption, partial consumption, or non-consumption of the menu offered to them.

Participants made a complete description of their dietary intakes such as the ingredients, cooking method, and brands to have better control over data processing. As support material, the interviewers used the “Tables of common home measures and habitual portion sizes for Spain population” [36] and the “Photo guide of food portions size of Spanish population” [37], including 204 food items regularly consumed by the Spanish population. Average daily Energy Intake (EI) (kcal) and macronutrient intakes (grams) were calculated using the software VD-FEN 2.1, a dietary evaluation program designed by the Spanish Nutrition Foundation (FEN), mainly based on data from a Spanish food composition table [38].

A total of 746 food items were recorded by the two 24 h DRs and were transformed into energy and nutrients for their analyses. Afterward, these items were grouped into 18 food groups to determine their contribution to the total Zn, Se, vitamin A, C, and E, and carotenoids (provitamin A: β-cryptoxanthin, α-carotene, and β-carotene; non-provitamin A: lutein, zeaxanthin, and lycopene) intakes: (1) milk and dairy products, (2) cereals, (3) meat and meat products, (4) oils and fats, (5) bakery and pastry, (6) fruits, (7) vegetables, (8) sugars and sweets, (9) ready to cook (industrial pre-cooked foods prepared with the expectation they will be heated/cooked by frying, microwave, oven, stovetop, or baked), (10) other dairy products, (11) beverages, (12) legumes, (13) eggs, (14) fish and shellfish, (15) appetizers, (16) cereal-based baby foods and supplements (cereal-based BFS), (17) nuts, and (18) sauces and condiments.

Since the average intake obtained by applying a 24 h dietary recall for a small number of days does not adequately represent the usual intake (UI), it was necessary to apply a statistical model to eliminate the day-to-day variation in food consumption. Therefore, the method developed by the Iowa State University (ISU) [39] was applied to remove the intra-individual variability and to obtain the individual UI distribution of Zn, Se, and vitamin C and E to determine the adequacy of recommendations. The ISU method was implemented using the PC-SIDE software (version 1.0, 2003, Iowa State University, Ames, IA, USA). For vitamin A, retinol, and carotenoids, the mean reported intake from the two 24 h DRs was calculated.

To assess nutrient adequacy, we used the DRVs from EFSA [33]. The EFSA included reference values for Zn, Se, and vitamins A, C, and E. The Adequate Intake (AI) was used for Se and vitamin E, and the Average Requirements (ARs) for Zn, vitamin A, and C. The proportion of subjects underneath the recommendations was calculated. Additionally, EFSA included a value of the tolerable upper intake levels (ULs) which is the maximum level of total chronic intake that could pose a risk of adverse health effects in humans [34]. Participants were divided into age groups established by EFSA for a full evaluation.

### 2.3. Sociodemographic and Anthropometric Data

Parents or caregivers were requested to complete a survey about general data regarding their children (e.g., age, sex, health status, etc.), sociodemographic data, and family backgrounds, such as parental education and occupation, family income (average monthly household income, in € ), employment, and lifestyle-related factors.

Children’s anthropometric measures (height/length and weight) were obtained by using their health card and then analyzed with the World Health Organization sex-specific growth charts, using Anthro and Anthro Plus software (WHO Anthro for personal computers, version 3.2.2, 2011).

### 2.4. Physical Activity and Sedentary Behavior

The physical activity and sedentary behavior questionnaire used in the EsNuPI study was a modification of a questionnaire previously validated in children aged <10 years from Colombia based on a seven-day recall [40]. Some modifications, such as language terms, were made to this questionnaire in order to adapt it to the needs of the present study. The protocol has been reported elsewhere [35].

### 2.5. Evaluation of Plausible Reporting and Misreporting (Under- and Over-Reporting)

Participants were identified as plausible, under-, or over-reporters of EI considering the relationship between their basal metabolic rate and their EI using Goldberg’s cut-offs, adapted for children [41]. Results from the EsNuPI study have been described previously [42].

Following the EFSA recommendations [43], the non-plausible reporters from the EsNuPI study were not excluded from the study subpopulations in the present analyses because the misreporting prevalence was low, and the exclusion of misreporters did not result in differences in nutrient intake.

### 2.6. Statistical Analysis

The Kolmogorov–Smirnoff normality test and histogram graphs were used to determine the normality of the distribution of the variables to decide between parametric or non-parametric analyses for comparisons. The variables of interest did not follow a normal distribution; therefore, we are reporting medians and IQR and non-parametric tests were performed.

The Mann–Whitney U test was applied to perform comparisons by total and age group between the subpopulations (REF and FMC). We also used the Kruskal–Wallis test to calculate differences among age groups within subpopulations. A chi-squared test was used to evaluate the differences in the percentages of adequacy between the subpopulations (REF and FMC) and among age groups within each subpopulation. The ANCOVA (analyses of covariance) was used to examine which variables could influence the intake of antioxidant minerals and vitamin intakes. Linear correlations, collinearity tests, and logistic regressions were performed to explore the possible role of various sociodemographic, anthropometric, and physical activity variables in antioxidant minerals and vitamin intakes. For these analyses, the 50th percentile was calculated in both subpopulations for each nutrient by sex and age group and then used to categorize children according to whether their intakes were below or above this cut-off point. The age group was used as the control variable in the analyzes. Statistical analyses were performed using IBM SPSS Statistics for Windows, version 20.0 (Armonk, NY, USA: IBM Corp.). A *p*-value less than 0.05 was considered as statistically significant.

## 3. Results

### 3.1. Subjects Characteristics

In Table 1, the personal, anthropometric, and socioeconomic characteristics of both study subpopulations by sex and age group are presented. In this table, we can observe that there are statistically significant differences between the REF and FMC groups in the variable Z-Height/Age for the total subpopulation and for boys.

### 3.2. Distribution of Total Antioxidant Minerals and Vitamins Intakes, and Adequacy to the Recommendations Set by EFSA

#### 3.2.1. Zinc

Table 2 shows that the median dietary intake of Zn ranged from 5.1 to 7.9 mg/day in the REF group and from 6.5 to 10.9 mg/day in the FMC group. When comparing the REF and FMC subpopulations by total Zn intake and age group, the children in the FMC group had a higher median Zn intake than REF. This trend was maintained in the three age groups (*p* < 0.001).

Differences in Zn intake among age groups of the same subpopulation were found. In the REF and FMC groups, the three age groups were statistically different (*p* < 0.001), with higher intakes as age increased.

According to the EFSA, the REF group comprised a higher percentage of children who met the AR recommendations for Zn than the FMC group (79.6% REF vs. 55.2% FMC). In Table 3, it can be observed that when comparing the percentages of adequacy by age group between subpopulations, we found that the three age groups from the REF group had a higher percentage of children below and meeting the EFSA recommendations in comparison with the FMC group, being statistically different (*p* < 0.001).

Nevertheless, in the three age groups, the FMC group showed a higher percentage of children (approximately three times or more than the REF group) exceeding the ULs proposed by the EFSA compared with the same age group of REF (*p* < 0.001).

#### 3.2.2. Selenium

Overall, the REF group had a higher median total Se intake when compared to the FMC group (61 µg/day REF vs. 51 µg/day FMC). This trend was repeated in the three age groups, and the differences were statistically significant (*p* < 0.005) (Table 2).

In terms of compliance with EFSA recommendations, the FMC group presented a higher percentage of children who met AI recommendations when compared to the REF group (87.1% REF vs. 90.8% FMC). Nevertheless, the REF group had a higher percentage of children above the ULs established for Se (11.5% REF vs. 7.7% FMC). This trend was repeated when comparing children from 3 to <6 years between the two subpopulations (Table 3).

#### 3.2.3. Vitamin A, Retinol, and Carotenoids

The results in Table 2 show statistically significant differences in vitamin A intake between the two subpopulations (*p* < 0.001). In the FMC group, vitamin A intakes were 636 µg retinol equivalents (RE)/day vs. 481 µg RE/day from REF. This pattern was repeated among the three age groups. Therefore, the FMC group had a higher percentage of children meeting EFSA recommendations (75.7% FMC vs. 71.3% REF) (*p* < 0.001) but also above the ULs (20.5% FMC vs. 13% REF) (*p* < 0.001) established for retinol. Furthermore, when compared to AR, the REF group had a higher percentage of children below the recommendations (10% REF vs. 3.8% FMC) (*p* < 0.001).

On the whole, vitamin A intake was higher in the FMC group, with a higher percentage of children meeting the AR and above the ULs. Therefore, the REF group had a higher percentage of children below both recommendations. Statistically significant differences were found in these results (*p* < 0.005).

The FMC group showed a higher total retinol intake than the REF group (376 µg RE/day FMC vs. 233 µg RE/day REF). Within all three age groups, the FMC group also had a higher retinol intake than the counterparts in the REF group. These differences were statistically significant (*p* < 0.001). Moreover, the REF group had a higher intake of total carotenoids than the FMC group (1079 µg/day REF vs. 998 µg/d FMC). Additionally, the children from 3 to <6 years in the REF group had a statistically significantly higher intake of carotenoids than those in the FMC group (1106 µg/d REF vs. 666 µg/d FMC) (*p* < 0.001) (Table 2).

#### 3.2.4. Vitamin C

Total vitamin C intake was significantly higher in the FMC group than in the REF group (114 mg/day FMC vs. 72 mg/day REF). The same results were observed when comparing by age group between subpopulations (Table 2). According to EFSA, it was observed that almost 100% of the children met the recommendations in both subpopulations (Table 3).

This characteristic was repeated for all age groups; however, a higher percentage of children below the recommendations was found in those aged 6 to <10 years in the REF group (7.0% REF vs. 0.5% FMC according to EFSA, *p* < 0.005).

#### 3.2.5. Vitamin E

According to Table 2, the total vitamin E intake in the FMC group was almost twice as high as in the REF group (8.9 mg/day vs. 4.5 mg/day, *p* < 0.005). These differences were found among all three age groups, which had significantly different intakes.

Inadequate intakes were observed in both subpopulations for vitamin E when compared to EFSA AIs. Dietary inadequacy was particularly high for vitamin E in the REF group, with 92.2% of the children below the AI, compared to the 33.3% shown in the FMC group. Hence, the FMC group showed the highest percentage of children meeting the AIs (66.7% FMC vs. 7.8% REF) (Table 3).

When comparing the three age groups between subpopulations, the percentage of the youngest children (1 to <3 years) in the FMC group meeting the EFSA AIs was significantly higher than that in the REF group (90.8% vs. 20.4%). More than 95% of children from 3 to <6 and 6 to <10 years in the REF group had inadequate vitamin E intakes, (3 to <6 years: 97.1% REF vs. 53.8% FMC; 6 to <10 years: 95% REF vs. 42.7% FMC).

Vitamin E intakes did not exceed the ULs in any of the children from the EsNuPI study.

### 3.3. Association between Usual Intakes of Antioxidant Minerals and Vitamins with Personal and Family Factors

Table 4 shows the socioeconomic variables that influenced the antioxidant minerals and vitamin intakes in both subpopulations of our study.

Supplementary Tables S1–S4 indicate the odds ratios (ORs) and confidence intervals (CIs) analyzing the total UI of antioxidant mineral and vitamin intakes and relative sociodemographic characteristics.

In the REF group, being a girl was associated with a 55% higher probability of having an intake ≥50th percentile (p50) for Se (*p* = 0.014). Moreover, living in a municipality with more than 300,000 inhabitants was associated with a lower probability of having intakes ≥p50 for Zn (OR = 0.47, 95% CI: 0.33–0.66, *p* < 0.001) and Se (OR = 0.65, 95% CI: 0.46–0.93, *p* = 0.017). Finally, having one parent who had completed secondary education was associated with a lower probability of having intakes ≥p50 for Se (OR = 0.55, 95% CI: 0.34–0.89, *p* = 0.016) and vitamin C (OR = 0.54, 95% CI: 0.35–0.82, *p* = 0.004), and children with one parent with university studies showed lower probabilities of intakes ≥p50 for vitamin A (OR = 0.70, 95% CI: 0.50–1.00, *p* = 0.050), vitamin C (OR = 0.54, 95% CI: 0.38–0.78, *p* < 0.001), and vitamin E (OR = 0.68, 95% CI: 0.48–0.97, *p* = 0.031). Children from 3 to <6 years old had a 53% and 58% higher probability of having an intake equal to or above the p50 for vitamins A and C, respectively. This group also showed lower probabilities of having intakes ≥p50 for Se (95%), Zn (92%), and vitamin E (40%). Moreover, children from 6 to <10 years had 54% and 70% lower probabilities of having intakes ≥p50 for Zn and Se, respectively (Supplementary Tables S1 and S2).

In the FMC group, the following aspects were associated with a higher probability of having intakes ≥p50 for Se: children drinking two or more bottles of milk per day (OR = 1.72 95%; CI: 1.12–2.66, *p* < 0.001), having one parent with university studies (OR = 1.52, 95% CI: 1.01–2.27, *p* = 0.044), the PAL (OR = 1.47 95%; CI:1.02–2.12, *p* = 0.041), and the children’s weight for age z-score (OR = 2.25, 95% CI: 1.33–3.79, *p* = 0.002). In contrast, living in a municipality of more than 300,000 inhabitants and belonging to a family with an income EUR ≥2000 was associated with a lower probability of having intakes ≥p50 for Se (OR = 0.69, 95% CI: 0.47–0.99, *p* = 0.046 and OR = 0.57, 95% CI: 0.33–1.00, *p* = 0.048, respectively) (Supplementary Table S3). Children who reported drinking two or more feeding bottles of milk per day in the sociodemographic questionnaire showed less probability of having intakes at or above the median value for vitamin A (OR = 0.43, 95% CI: 0.30–0.62, *p* < 0.001) and vitamin C (OR = 0.53, 95% CI: 0.37–0.77, *p* < 0.001). PAL reduced the odds of having a vitamin A intake equal to or greater than the median (OR = 0.43, 95% CI: 0.30–0.62, *p* < 0.001). Additionally, for vitamin C, having one parent with secondary education (OR = 0.63, 95% CI: 0.41–0.97, *p* = 0.037) as well as children’s height for age z-score (OR = 0.61, 95% CI: 0.40–0.93, *p* = 0.022) were associated with a lower probability of having intakes ≥p50 (Supplementary Table S4).

Finally, also for the FMC group, children between 3 to <6 years old had a 62% higher probability of having intakes ≥p50 for vitamin A. On the contrary, regarding Zn and Se, children from this age group showed 94% and 96% lower probabilities of having intakes ≥p50. Children from 6 to <10 years old showed a lower likelihood of having intakes equal to or above p50 for Zn (62%) and Se (77%) (Supplementary Table S3).

### 3.4. Food Groups Contributing to Antioxidant Minerals and Vitamins Intakes

**Figure 1:**
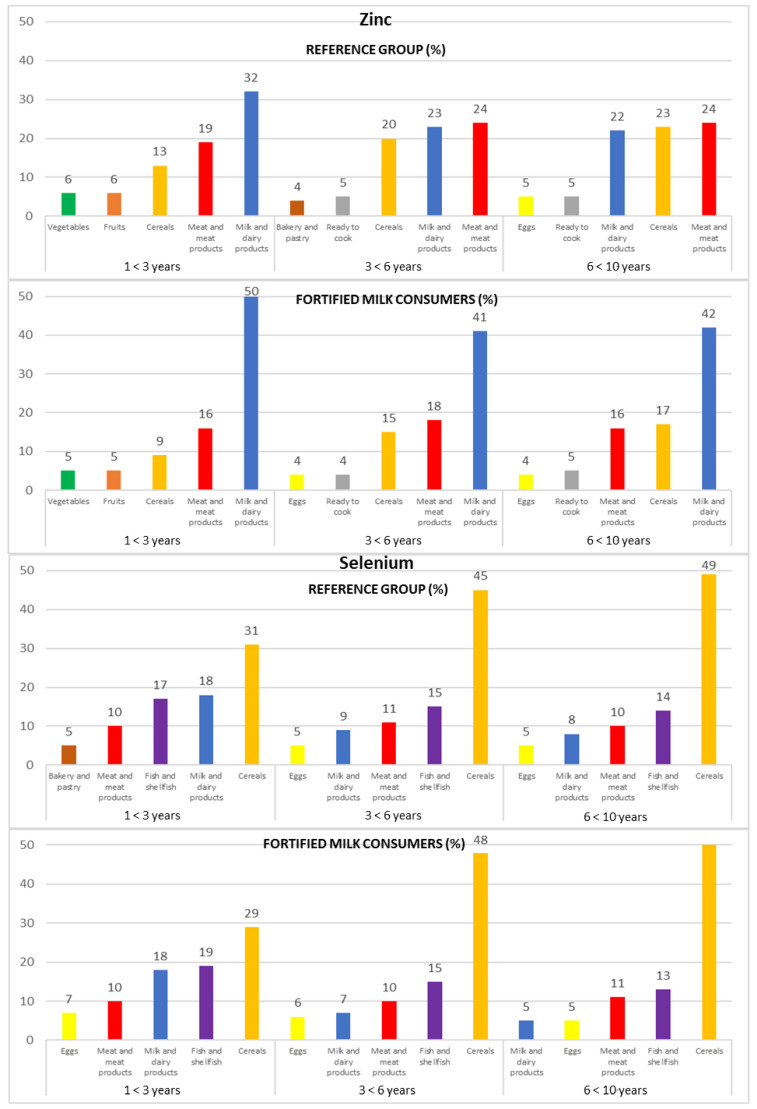
Contribution of the main five food groups (in percentages) to total zinc and selenium intakes in the EsNuPI study subpopulations according to age group: 1 to <3 years, 3 to <6 years, and 6 to <10 years.

**Figure 2:**
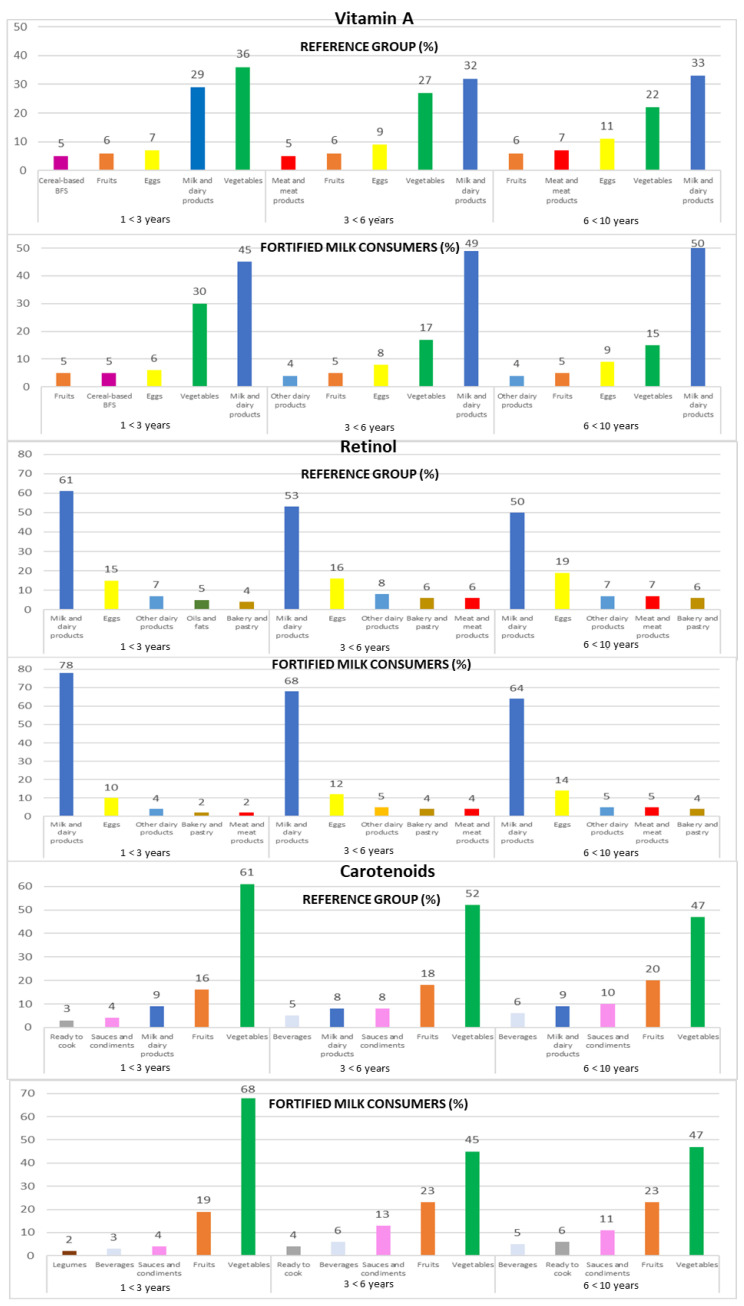
Contribution of the main five food groups (in percentages) to total vitamin A, retinol, and carotenoids intakes in the EsNuPI study subpopulations according to age group: 1 to <3 years, 3 to <6 years, and 6 to <10 years. Cereal-based baby food and supplements (cereal-based BFS).

**Figure 3:**
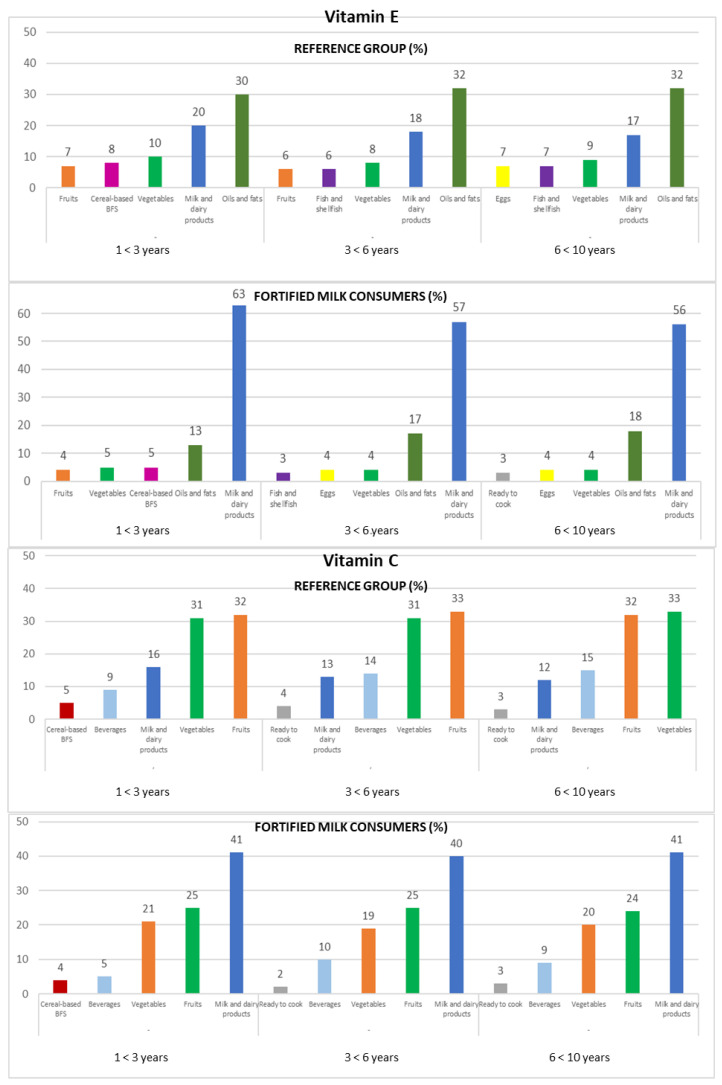
Contribution of the main five food groups (in percentages) to total vitamin E and C intakes in the EsNuPI study subpopulations according to age group: 1 to <3 years, 3 to <6 years, and 6 to <10 years. Cereal-based baby food and supplements (cereal-based BFS).

Intakes from food and beverage sources of selected antioxidant minerals and vitamins are presented in Figure 1, Figure 2 and Figure 3, respectively.

#### 3.4.1. Zinc

The main sources of Zn for both subpopulations were milk and dairy products (25% REF and 50% FMC), meat and meat products (23% REF and 17% FMC), and cereals (20% REF and 13% FMC).

Following this trend when analyzing by age groups, the same food groups represented the most important sources of Zn in the three age groups of both subpopulations (REF and FMC, respectively): milk and dairy products (1 to <3 years: 32% and 50%; 3 to <6 years: 23% and 41%; 6 to <10 years: 22% and 42%), meat and meat products (1 to <3 years: 19% and 16%; 3 to <6 years: 24% and 18%; 6 to <10 years: 24% and 16%), and cereals (1 to <3 years: 13% and 9%; 3 to <6 years: 20% and 15%; 6 to <10 years: 23% and 17%) (Figure 1).

#### 3.4.2. Selenium

The cereals food group (44% REF and 41% FMC) was the main source of Se intakes in both subpopulations, followed by fish and shellfish (15% REF and 16% FMC), milk and dairy products (11% in both subpopulations), and meat and meat products (10% in both subpopulations). These food groups provided about 85% of Se intake.

In addition, in both subpopulations (REF and FMC, respectively), we can observe that cereals increase their Se contribution with increasing age (1 to <3 years: 31% and 29%; 3 to <6 years: 45% and 48%; 6 to <10 years: 49% and 51%); contrary to the above-mentioned results, fish and shellfish (1 to <3 years: 17% and 19%; 3 to <6 years: 15% in both subpopulations; 6 to <10 years: 14% and 13%) and milk and dairy products (1 to <3 years: 18% in both subpopulations; 3 to <6 years: 9% and 7%; 6 to <10 years: 8% and 5%) decrease their contribution to this mineral as age increases.

Meat and meat products intake remained stable in the REF and FMC group (1 to <3 years: 10% in both subpopulations; 3 to <6 years: 11% and 10%; 6 to <10 years: 10% and 11%, respectively) (Figure 1).

#### 3.4.3. Vitamin A

Milk and dairy products were recorded as the predominant source of vitamin A (32% REF and 48% FMC), followed by vegetables (27% REF and 22% FMC) and eggs (9% REF and 7% FMC). The top three sources of vitamin A remained constant across the three age groups for both subpopulations (REF and FMC, respectively): milk and dairy products (1 to <3 years: 29% and 45%; 3 to <6 years: 32% and 49%; 6 to <10 years: 33% and 50%), vegetables (1 to <3 years: 36% and 30%; 3 to <6 years: 27% and 17%; 6 to <10 years: 22% and 15%), and eggs (1 to <3 years: 7% and 6%; 3 to <6 years: 9% and 8%; 6 to <10 years: 11% and 9%) (Figure 2).

#### 3.4.4. Retinol

The leading sources of retinol were milk and dairy products (54% REF and 71% FMC), followed by eggs (17% REF and 12% FMC) and other dairy products (7% REF and 5% FMC). This same food groups contributed to retinol intakes when analyzing data by age groups in both subpopulations (REF and FMC, respectively): milk and dairy products (1 to <3 years: 61% and 78%; 3 to <6 years: 53% and 68%; 6 to <10 years: 50% and 64%), eggs (1 to <3 years: 15% and 10%; 3 to <6 years: 16% and 12%; 6 to <10 years: 19% and 14%), and other dairy products (1 to <3 years: 7% and 4%; 3 to <6 years: 8% and 5%; 6 to <10 years: 7% and 5%) (Figure 2).

#### 3.4.5. Carotenoids

Children mainly obtained their carotenoid intakes from vegetables (52% REF and 55% FMC), fruits (18% REF and 21% FMC), and milk and dairy products (9% REF and 3% FMC). When analyzing data by the three age groups from both subpopulations, the same food groups contribute to carotenoid intakes (REF and FMC, respectively): vegetables (1 to <3 years: 61% and 68%; 3 to <6 years: 52% and 45%; 6 to <10 years: 47% in both subpopulations), fruits (1 to <3 years: 16% and 19%; 3 to <6 years: 18% and 23%; 6 to <10 years: 20% and 23%), milk and dairy products (1 to <3 years: 9% and 1%; 3 to <6 years: 8% and 4%; 6 to <10 years: 9% and 3%), and sauces and condiments (1 to <3 years: 4%; 3 to <6 years: 8% and 13%; 6 to <10 years: 10% and 11%) (Figure 2).

#### 3.4.6. Vitamin C

Vitamin C intakes were mainly provided by fruits (32% REF and 25% FMC), vegetables (32% REF and 20% FMC), and milk and dairy products (13% REF and 41% FMC), accumulating 76% (REF) and 86% (FMC) of the total vitamin C intakes (Figure 3).

The ranking changes slightly when analyzed by the three age groups. On the one hand, in the REF group, the fruits (1 to <3 years: 32%; 3 to <6 years: 33%; 6 to <10 years: 32%) represented the main source of this vitamin, followed by vegetables (1 to <3 years and 3 to <6 years: 31%; 6 to <10 years: 33%) and milk and dairy products (1 to <3 years: 16%; 3 to <6 years: 13%; 6 to <10 years: 12%); on the other hand, the milk and dairy products food group (1 to <3 years: 41%; 3 to <6 years: 40%; 6 to <10 years: 41%) was the highest source of vitamin C in the FMC group, followed by fruits (1 to <3 years and 3 to <6 years: 25%; 6 to <10 years: 24%) and vegetables (1 to <3 years: 21%; 3 to <6 years: 19%; 6 to <10 years: 20%) (Figure 3).

#### 3.4.7. Vitamin E

In the REF group, milk and dairy products contributed 20% to their vitamin E intake. Nevertheless, they provided about 60% of the vitamin E intake in the FMC group. In both subpopulations, the oils and fats (32% REF and 15% FMC) and vegetables (9% REF and 4% FMC) contributed to vitamin E intake.

Amongst the three age groups, in the FMC group, milk and dairy products (1 to <3 years: 63%; 3 to <6 years: 57%; 6 to <10 years: 56%), oils and fats (1 to <3 years: 13%; 3 to <6 years: 17%; 6 to <10 years: 18%), and vegetables (1 to <3 years: 5%; 3 to <6 years and 6 to <10 years: 4%) were the main sources of vitamin E. In the REF group, oils and fats were the main food sources of vitamin E (1 to <3 years: 30%; 3 to <6 years and 6 to <10 years: 32%), followed by milk and dairy products (1 to <3 years: 20%; 3 to <6 years: 18%; 6 to <10 years: 17%) and vegetables (1 to <3 years: 10%; 3 to <6 years: 8%; 6 to <10 years: 9%) (Figure 3).

## 4. Discussion

This work provides recent estimates of the distribution of antioxidant mineral (Zn and Se) and vitamin (vitamin A, C, and E) intake and food sources in one representative subpopulation of Spanish children aged 1 to <10 years who consumed all types of milk and another with the same characteristics that consumed FMF. In addition, the effect of different sociodemographic factors (sex, education level, family income, etc.) on the antioxidant intakes was evaluated.

Our results highlight that many children in both subpopulations show high intakes of Zn, Se, and vitamin C. However, it is also of great importance to know that many children have inadequate vitamin A and vitamin E intakes. This fact stands out in the age group 6 to <10 years, which shows a large percentage of children who are below the recommendations for these two nutrients. Similar results were found in children aged nine years and older in the ENALIA study [25]. This trend among Spanish children could be attributed to the current low adherence to the traditional Mediterranean Diet [45]. Likewise, the present study revealed that the major dietary sources contributing the most antioxidant micronutrients were milk and dairy products, meat and meat products, cereals, vegetables, and fruits.

Regarding the association between sociodemographic factors and intakes of antioxidant minerals and vitamins in the REF group, we found that the factor that most influenced the intake of Zn, Se, and vitamin E was the children’s age: being 3 to < 6 years old appeared to be associated with the possibility of being below the median value of the aforementioned antioxidants. On the contrary, being between 3 and <6 years old increases the likelihood of being above the median intake of vitamins A and C. In addition, having a parent with university studies was associated with lower probabilities of having intakes below the median values for Se and vitamin C.

Our results are in agreement with the findings reported by others. In the Brazilian Health Survey Sao Paulo (*n*= 1511, aged 14–97 years), the results showed that some age/sex groups were associated with a lower intake of Zn and Se. In addition, having a household income above the minimum wage was positively associated with Se intake [46].

This study showed that, in general, Zn intakes were above the DRV (AR) in both subpopulations; in addition, the REF group showed 24% more children meeting the recommendations for this mineral than in the FMC group; at the same time, the REF group showed approximately 10% more children with an intake at risk of being inadequate compared to the FMC group, especially in the age groups 1 to <3 and 6 to <10 years. In addition, more than 40% of children in the FMC group exceeded the ULs for Zn, and although there is little risk of adverse effects in children who exceed ULs by a modest amount, the question of a potential impact on iron and copper absorption may be raised.

Severe Zn deficiency is uncommon in European populations, and marginal deficiency is likely to be more prevalent [47,48]. When comparing our data with other studies, we observed that the Zn intake reported by our study subjects was similar to that reported by the 1-to-13-year-old children from the ENALIA project (8.7 mg/day) [25]. In the ANIBES study, 31% of the children aged 9 to 12 years had an inadequate intake of Zn according to the EFSA recommendations [26]. The NHANES study reported 8.5 mg/day as UI of Zn [27]; this result is higher than the 6.5 mg/day of our REF group. In addition, low Zn intake was reported in 56% of French children consuming milk and milk-based products, and 33% of those were consuming FMF [49].

Zn is primarily found in animal products and seafood, which is why the main group that contributed to this mineral intake was milk and dairy products, followed by meat and meat products, and the food group “cereals”. Low fish consumption could explain the insufficient intake of Zn. In the ANIBES study, the order of importance in the contribution was different; meat and meat products, cereals, and milk and milk products provided 70% of Zn intake [26]. In Australian children (*n*= 4834; 2–16 years), meat and poultry, milk products, cereals, and cereal products contributed 68% of total Zn intake [50].

In all studied groups, the reported intake of Se was practically in compliance with EFSA recommendations, with only about 2% of children below the recommendations in both subpopulations. In the ENALIA study, 5% of children were below the EAR and 10% were above the Uls [25]. The ENALIA and ANIBES studies reported a Se intake higher than our study (mean 83 µg/day and 69 µg/day, respectively). In addition, the Se intake reported by children in the EsNuPI study was lower than that reported in Spanish children of 8 to 13 years [51] and in children from other European countries [52].

The main sources of Se in our study population were the following food groups: cereals, fish and shellfish, milk and dairy products, and meat and meat products. The cereals food group contributed a high amount (more than 40% in both subpopulations) to the intake of this mineral because their consumption was also high; fish and shellfish contributed about 15% in both subpopulations; however, in this case, consumption has decreased significantly in Spain, as demonstrated in previously published studies [42]. In agreement with our study, in the ANIBES study, the two main sources of Se were cereals and grains (46.5%) and fish (16.7%) [26].

Vitamin A deficiency is one of the most common childhood health problems in developing countries, leading to visual problems, growth retardation, etc. The term vitamin A includes provitamin A carotenoids that are dietary precursors of retinol. However, to date, no adequate intake of carotenoids has been established for children. Nevertheless, low concentrations of serum retinol have been found in overweight and obese individuals [53].

Our study showed that vitamin A intakes were adequate in both subpopulations, with approximately more than 70% of the children meeting the EFSA recommendations. Nevertheless, the REF group encompassed a higher percentage of children below the EFSA recommendations. It is important to mention that as age increases, the percentage of children that do not meet the recommendations does as well. Our results are in agreement with a study conducted on Greek children aged 1–19 years that showed a considerable percentage of subjects with insufficient intakes of vitamin A, especially in children 9 to 19 years (~50%) [54].

In the ALSALMA study, children 1 to 3 years old had a vitamin A intake similar to the reported in children 1 to <3 years from our FMC group (775 µg RE/day and 756 µg RE/day, respectively), and higher than our REF group (576 µg RE/day) [24]. Comparing these data with the ENALIA study, we observed that vitamin A intakes were higher (856 µg RE/day) than those reported by our studied children [25]. In the NHANES study, only 1.3% of children were below the EAR [27]; this is a very low percentage when compared to our results. Like what was observed in the EsNuPI FMC group, the children from the Childhood Obesity Project (3 months up to 8 years old) from European countries had adequate vitamin A intake at all time points, with 95% of the children meeting or being above the EAR [55].

Milk and dairy products were the main sources of vitamin A in the EsNuPI study, preceding vegetables, eggs, and fruits. It is important to underline that approximately 45% of vitamin A was provided by the milk and dairy products consumed by the FMC group, which were about 15% higher than in the REF group. In the ANIBES study, vegetables were the main source of vitamin A, followed by milk and dairy products, eggs, and fruit [26]. Likewise, data from 12 dietary surveys in nine European Union countries concluded that the main sources of vitamin A were vegetables, milk, and dairy products [56].

The children from the EsNuPI study have an adequate vitamin C intake when referring to the AR established by the EFSA, with more than the 95% of the children meeting the recommendations. The reported vitamin C intake in our study is higher than that reported in the ANIBES study (with 36% not meeting the EFSA recommended intakes) but lower than data from the Nutri-bebé survey (*n* = 1035; French children aged 0.5–35 months), in which the median vitamin C ranged from 4 to 255 mg/day [26,57].

Almost 90% of vitamin C comes from vegetables and fruits. Humans are unable to synthesize it, making vitamin C an essential dietary micronutrient [58,59]. Consistent with our findings, vegetables, fruits, milk, and dairy products were the main food sources of vitamin C in the ANIBES study [26]. Previous studies on Greek children have found fruit (17.6%) and fruit juices (16.8%) ranked among the contributors to total vitamin C intake [54].

Vitamin C is essential for preventing scurvy in humans and is involved in the prevention of diseases such as coronary heart disease, stroke, and cancer. Evidence indicates that deficiency and hypovitaminosis of this vitamin are common in low- and middle-income countries and not uncommon in high-income countries [60,61,62].

For many children in the present survey, vitamin E intakes were below the EFSA guidelines, especially in the REF group. Our study highlights the poor mean vitamin E intake and the fact that the prevalence of inadequacy was over 90% according to EFSA across all age groups in the REF group. Only 9.4% of the children meet the recommendations in the REF group, while 64.7% meet them in the FMC group. The results in the REF group are in accordance with the figure of 63% of the population being below the DRIs for vitamin E reported by the Valencian Anthropometry and Child Nutrition (ANIVA) study with Spanish children 6 to 9 years old [63]. The prevalence of inadequacy reported was similar to that reported by the NHANES study; children aged 1 to 6 years had a vitamin E intake of 4.9 mg/day, hence why 69% of the children had intakes below the EAR [27]. These results were in accordance with those shown in the REF group, which had an intake of 4.8 mg/day, and 90% of the children were below the EFSA chourecommendations. Similar data were observed in Spanish adolescents in the ENALIA study, who showed a vitamin E intake of 8.83 mg/day in children from 1 to 13 years [25].

Vitamin E deficiency is more frequently found in children than in adults due to low body reserves and intense growth and development, making the low vitamin E intake reported in the current study a major concern. The importance of vitamin E is underlined by its antioxidant activity and its potential protective role against cardiovascular disease, certain types of cancer, and neurological diseases. Nevertheless, 91%, 46%, and 57% of the children in the three age groups from the FMC group meet EFSA recommendations; this may be explained by the fact that some FMFs are fortified with vitamin E. These results were in agreement with results shown by Chouraqui et al., 2019 [57].

Numerous foods provide vitamin E; primarily, nuts, seeds, vegetable oils, and fatty fish are the best sources of alpha-tocopherol. Almost sixty percent of vitamin E in the FMC group and around 20% in the REF group came from milk and dairy products; oils and fats (including olive oil) represent the main source of this vitamin in REF (~31%) and the second source in the FMC group (~16%). However, in the ANIBES study, the main contributors to vitamin E intake were oils and fats, followed by vegetables [26]. In Greek children and adolescents, salty snacks were the main source of vitamin E (12.5%) [54]. Different studies attribute the high percentage of insufficient vitamin E intake to the low consumption of whole grains, seeds, and oilseeds [64]. One possible cause of the low vitamin E intake could be due to the fact that in Spain, the consumption of traditional foods from the Mediterranean Diet, such as olive oil and legumes, has decreased notably in recent years among the young population [42,45,65].

In general, low dietary intakes of antioxidant minerals and vitamins and a diet low in fruit and vegetables predict increased OS in adolescents and adults [66,67,68]. Increased OS is associated with metabolic risk factors and may contribute to the development of different comorbidities, including type 2 diabetes, cardiometabolic diseases, and atherosclerosis [8,69,70,71]. In addition, it has been found that similar to obese adults, obese children show a higher degree of OS than normal-weight children [72,73]. Faienza and cols., 2012 [74] demonstrated the role of obesity in altering oxidant–antioxidant status in children, whereby obese children during prepubertal age may develop metabolic abnormalities that can lead to metabolic syndrome and cardiovascular heart disease.

Moreover, obesity has been associated with decreased plasma levels of antioxidant vitamins and antioxidant capacity [72]. Therefore, scientific evidence suggests that antioxidant nutrients may be an important factor in the treatment and prevention of obesity and its comorbidities [47,72]. The bioactive compounds in the diet possess antioxidant activities for the normal function of the human organism, which can be protective against chronic diseases [75,76,77,78,79]. The Mediterranean Diet provides all the macro and micronutrients necessary to keep an organism in optimal balance and counteract oxidative damage, but changes in the diet of Spanish children in the last few decades have led to changes in dietary patterns, moving away from this traditional pattern to adopt a less healthy diet with a high intake of palatable energy-dense foods and the lower consumption of vegetables and fruits [45,80,81,82].

In this study, it is observed that the intake of antioxidant minerals and vitamins is higher in the FMC than in the REF group. Therefore, the use of FMF could help to meet the recommendations established by the EFSA. Several studies have been conducted to compare the nutrient intakes of children consuming FMF and CM. In a French study in 1–2-year-old children, FMF consumers had a significantly reduced risk of insufficiency of Zn (4.6 mg/day CM and 6.4 mg/day FMF), vitamin A (788 µg/day CM and 1119 µg/day FMF), C (52 mg/day CM and 82 mg/day FMF), and E (2.7 mg/day CM and 6.2 mg/day FMF), compared to those consuming CM [42].

A simulation study investigated the nutritional role of FMF and found that children 1 to 3 years old that consumed FMF increased their total dietary intake of iron and vitamins A, B, C, and E and had a lower risk of insufficient micronutrient intake [83]. Children who changed consumption from CM to FMF decreased the percentage that was not meeting the EARs. It decreased from 26 to 11% vitamin A, 53 to 27% vitamin C, and 54 to 24% vitamin E [83].

A recent clinical trial conducted in Australia also demonstrated that FMF consumption was associated with increased nutritional adequacy and a greater likelihood of meeting nutrient requirements such as Zn, Fe, vitamins D, and C in children aged 1–2 years compared to the unfortified CM group [33]. Similar findings were also reported by Walton and Flynn in Irish children aged 1–2 years with intakes of Zn (5.1 mg/day non-FMF and 7.3 mg/day FMF), iron (5.9 mg/day and 10.4 mg/day FMF), vitamins A (759 µg/day non-AM and 969 µg/day FMF), vitamin C (58 mg/day non-FMF and 118 mg/day FMF), and vitamin D (2.1 µg/day non-FMF and 9.2 µg/day FMF) [84]. Likewise, in Chinese children aged 1–3 years, the consumption of FMF contributes to improving the nutrient intakes of vitamin A (457µg/day CMs and 730 µg/day FMF), vitamin C (47 mg/day CMs and 65 mg/day FMF), vitamin D (11.3 µg/day CMs and 17.2 µg/day FMF), and vitamin E (9.4 mg/day CMs and 12.0 mg/day FMF) [85]. Finally, our findings are in accordance with a study conducted in Filipino children aged 1–4 years, in which it was reported that AM consumers were more likely to achieve adequacy of Zn, vitamin B_6_ and B_12_, vitamin C, and vitamin D compared to other milk consumers [86].

### Strengths and Limitations

The main strength of the EsNuPI study is that it is the first study to analyze a subpopulation that is representative of Spanish children aged 1 to <10 years and a subpopulation of children of the same age who consume FMF, a type of milk that is increasingly consumed in Spanish households. Additionally, the methodologies for the design, collection, and processing of the data were developed following the recommendations of different official entities; for instance, to obtain the information through a 24 h DR, the methodology recommended by the EFSA was followed.

The present study is subjected to the following limitations: (1) errors in the reported information might have influenced the results of the study questionnaires. Hence, following the EFSA recommendations, under- and over-reporting were identified in this study and analyzed separately; (2) to obtain data representative of a subject’s diet, several 24 h DRs are necessary. Therefore, applying Nusser’s method, nutrient intakes were transformed into IU to eliminate intraindividual variability, which allowed a better estimation of the distribution of nutrient intakes [39]; (3) we studied children living in urban areas, and this could be considered as a potential limitation; however, currently, 52.6% of the total Spanish population from 1 to <10 years old live in urban areas; (4) due to the study’s non-invasive design, plasma antioxidant vitamin levels were not available for analyses; (5) our survey enquired about child’s supplement use (only 2.9% reported the consumption of any type of supplement, compared to the 7–25% reported for Spanish children [25]). Supplements reported in the 24 h DR were included in the “cereal-based BFS” food group.

## 5. Conclusions

This study aimed to provide information on the intakes of antioxidant micronutrients (Se, Zn, vitamin A, C, and E) among children aged 1 to <10 years from urban areas in Spain. Although findings indicate that UIs of Zn and Se were adequate amongst infants and children, a significant proportion of them showed Zn (10.2% REF and 2.0% FMC) deficiency. Furthermore, a high percentage of children had vitamin A intakes below the recommendations (15.7% REF and 3.8% FMC) and vitamin E intakes (92.2% REF and 33.3% FMC). The low intakes of vitamin E reported in this study are of great importance to stakeholders.

The main sources of Zn, vitamin A, vitamin E, and vitamin C were milk and dairy products in both subpopulations. Moreover, children consuming FMF were more likely to meet the reference ranges established by EFSA for antioxidant nutrients. It is necessary to improve the strategies to increase the consumption of antioxidant minerals and vitamins, in addition to providing appropriate nutrition education for caregivers.

Further research is needed to evaluate the dietary intakes of a higher number of antioxidant minerals and vitamins and to also assess biomarkers of antioxidant nutrient intake for accurate measurement and association with diverse factors of oxidative damage.

## Figures and Tables

**Table 1:** Sociodemographic and anthropometric characteristics by age group and sex of two subpopulations, differing based on milk intake.

		Reference Group (REF)			Fortified Milk Consumers (FMCs)		
		Total	Boys	Girls	Total	Boys	Girls
		*n* = 707	*n* = 357	*n* = 350	*n* = 741	*n* = 371	*n* = 370
Age, mean ± SD (years)	1 to <3 years	1.52 ± 0.50	1.60 ± 0.49	1.44 ± 0.50	1.46 ± 0.50	1.44 ± 0.50	1.48 ± 0.50
	3 to <6 years	3.87 ± 0.82	3.85 ± 0.82	3.89 ± 0.83	3.79 ± 0.82	3.81 ± 0.83	3.76 ± 0.82
	6 to <10 years	7.60 ± 1.12	7.55 ± 1.11	7.66 ± 1.12	7.57 ± 1.10	7.61 ± 1.11	7.53 ± 1.09
	1 to <3 years	162 (22.9) *	84 (23.5) *	78 (22.3) *	294 (39.7) *	144 (38.8) *	150 (40.5) *
Age group, *n* (%)	3 to <6 years	244 (34.5) *	122 (34.2) *	122 (34.9) *	262 (35.4) *	128 (34.5) *	134 (36.2) *
	6 to <10 years	301 (42.6) *	151 (42.3) *	150 (42.9) *	185 (25) *	99 (26.7) *	86 (23.2) *
Anthropometric characteristics, median (IQR)	Z-BMI/Age	0.6 (−0.3–1.5)	0.6 (−0.3–1.5)	0.6 (−0.3–1.4)	0.5 (−0.3–(−1.4)	0.45 (−0.3–1.4)	0.5 (−0.3–1.4)
	Z-Weight/Age	0.5 (−0.3–1.2)	0.4 (−0.4–1.2)	0.6 (−0.3–1.3)	0.6 (−0.3–1.4)	0.6 (−0.1–1.4)	0.5 (−0.3–1.4)
	Z-Height/Age	−0.3 (−1.2–(−0.9))	−0.2 (−1.1–1.0)	−0.4 (−1.3–0.7)	−0.4 ** (−1.4–0.6)	−0.4 ** (−1.4–0.6)	−0.4 (−1.5–0.6)
PAL, median (IQR)	1 to <3 years	1.6 (1.3–1.8)	1.6 (1.4–1.8)	1.5 (1.3–1.8)	1.5(1.3–1.7)	1.5 (1.3–1.8)	1.5(1.3–1.7)
	3 to <6 years	1.6 (1.4–1.7)	1.6 (1.4–1.7)	1.5 (1.4–1.7)	1.5 (1.4–1.7)	1.5(1.4–1.7)	1.5(1.4–1.7)
	6 to <10 years	1.6 (1.4–1.7)	1.6 (1.4–1.8)	1.6 (1.5–1.7)	1.6 (1.5–1.7)	1.6(1.5–1.8)	1.6(1.5–1.7)
Size of the municipality, *n* (%)	50.001 a 300.000 people	376 (53.2)	193 (54.1)	183 (52.3)	406 (54.8)	204 (55.0)	202 (54.6)
	>300.000 people	331 (46.8)	164 (45.9)	167 (47.7)	335 (45.2)	167 (45.0)	168 (45.4)
Highest level of education achieved by one of the parents, *n* (%)	≤10 years of education	23 (3.3)	10 (2.9)	13 (3.8)	14 (1.9)	7 (1.9)	7 (1.9)
	Secondary education	416 (60.5)	219 (62.9)	197 (57.9)	414 (57.0)	208 (57.5)	206 (56.6)
	University studies	249 (36.2)	119 (34.2)	130 (38.2)	298 (41.0)	147 (40.6)	151 (41.5)
Family income, *n* (%)	Low (<1500 EUR)	171 (24.2)	79 (22.1)	92 (26.3)	163 (22.0)	84 (22.6)	79 (21.4)
	Medium (1501 to 2000 EUR)	126 (17.8)	67 (18.8)	59 (16.9)	134 (18.1)	64 (17.3)	70 (18.9)
	High (>2000 EUR)	226 (32.0)	123 (34.5)	103 (29.4)	238 (32.1)	110 (29.6)	128 (34.6)
	No answer/doesn’t know	184 (26.0)	88 (24.6)	96 (27.4)	206 (27.8)	113 (30.5)	93 (25.1)
Number of feeding bottles or glasses of milk per day, *n* (%)	Less than 2	222 (32.9)	110 (32.0)	115 (33.8)	178 (24.1)	92 (24.9)	86 (23.3)
	2 o more	459 (67.1)	234 (68.0)	225 (66.2)	561 (75.9)	278 (75.1)	283 (76.7)

BMI: Body Mass Index; PAL: Physical Activity Level. The PAL was calculated for individual and group level according to the European Food Safety 269 Authority (EFSA) protocol to assess misreporting [22]. Values are presented as median (interquartile range) or percentage per group. * Significant differences between the reference group and fortified milk consumers (in the total and by sex) are shown, applying the Chi-square and Mann–Whitney tests. ** *p* < 0.01 difference vs. reference group (Mann–Whitney’s U test). Adapted with permission from Ref. [44].

**Table 2:** Total antioxidant minerals and vitamins intakes by age and subpopulations (*n* = 1448).

**Reference Group (REF)**							
	**1 to <3 Years** ***n* = 162**		**3 to <6 Years** ***n* = 244**		**6 to <10 Years** ***n* = 301**		
	**Median**	**IQR**	**Median**	**IQR**	**Median**	**IQR**	***p***
Zinc (mg/day)	5.1 ^a^	2.1	6.9 ^b^	1.7	7.9 ^c^	2.8	<0.001
Selenium (µg/kg)	38 ^a^	27	58 ^b^	19	70 ^c^	21	<0.001
Vitamin A (µg/day) ~	495	401	499	400	450	391	0.473
Retinol (µg RE/day) ~	202 ^a^	166	238 ^b^	184	242 ^b^	233	<0.001
Carotenoids (µg/day) ~	1383 ^a^	2000	1106 ^a^	1541	743 ^b^	1304	<0.001
Vitamin C (mg/day)	78 ^a^	44	69 ^b^	33	71 ^ab^	42	0.021
Vitamin E (mg/day)	3.9 ^a^	2.4	4.7 ^b^	2.4	4.7 ^b^	2.6	0.002
**Fortified Milk Consumers (FMCs)**							
	**1 to <3 years** ***n* = 162**		**3 to <6 years** ***n* = 244**		**6 to <10 years** ***n* = 301**		
	**Median**	**IQR**	**Median**	**IQR**	**Median**	**IQR**	***p***
Zinc (mg/day)	6.5 ^a^*	2.1	8.6 ^b^*	3.1	10.9 ^c^*	5.0	<0.001
Selenium (µg/kg)	37 ^a^	20	54 ^b^*	15	67 ^c^*	20	<0.001
Vitamin A (µg/day) ~	693 *	360	596 *	359	605 *	327	0.473
Retinol (µg RE/day) ~	368 ^a^*	179	372 ^b^*	198	410 ^b^*	284	<0.001
Carotenoids (µg/day) ~	1492 ^a^	1879	666 ^a^*	1384	763 ^b^	1049	<0.001
Vitamin C (mg/day)	119 ^a^*	33	107 ^b^*	49	110 ^ab^*	56	0.021
Vitamin E (mg/day)	8.4 ^a^*	2.8	8.9 ^b^*	3.5	9.6 ^b^*	3.9	<0.001

(~) Data are presented as the average intake values from two 24 h DRs for vitamin A, retinol, and carotenoids. The Usual Intakes were calculated for zinc, selenium, and vitamin C. Mann–Whitney U-test was used to evaluate differences by age group between REF and FMC groups (significant differences are marked with an asterisk (*) symbol in median values of the FMC). Kruskal–Wallis test was used to calculate differences among age groups within subpopulations (significant differences are marked with superscript letters in the median values of each age group). *p*-values for this test are included in the last column. *p*-value < 0.05 was considered statistically significant.

**Table 3:** Adequacies to the European Food Safe Authority (EFSA) recommendations (AR and AI) for antioxidants minerals and vitamins by subpopulation and age group (*n* = 1448).

**Reference Group (REF)**										
	**1 to <3 Years** ***n* = 162**			**3 to <6 Years** ***n* = 244**			**6 to <10 Years** ***n* = 301**			
	**% <AR**	**% >AR**	**% >UL**	**% <AR**	**% >AR**	**% >UL**	**% <AR**	**% >AR**	**% >UL**	***p***
Zinc	14.8	72.2	13.0	1.2	82.0	16.8	14.6	81.7	3.7	<0.001
Selenium	5.6	80.9 ^a^	13.6	0	79.1 ^a^	20.9	0.3	97.0 ^b^	2.7	<0.001
Vitamin A	10.5	68.5	21.0	10.2	75.4	14.3	22.9	69.4	7.6	<0.001
Vitamin C	1.2	98.8 ^a^	0	0.0	100 ^a^	0	7.0	93.0 ^b^	0	<0.001
Vitamin E	79.6	20.4 ^a^	0	97.1	2.9 ^b^	0	95.0	5.0 ^b^	0	<0.001
**Fortified Milk Consumers (FMCs)**										
	**1 to <3 years** ***n* = 294**			**3 to <6 years** ***n* = 262**			**6 to <10 years** ***n* = 185**			
	**% <AR**	**% >AR**	**% >UL**	**% <AR**	**% >AR**	**% >UL**	**% <AR**	**% >AR**	**% >UL**	***p***
Zinc	0.7 *	63.9 ^a^	35.4 *	1.1	43.5 *^b^	55.3 *	4.3 *	57.8 *^a^	37.8 *	<0.001
Selenium	3.4	87.4 ^a^	9.2	0	90.1 *^a^	9.9 *	0.5	97.3 ^b^	2.2	<0.001
Vitamin A	0.7 *	65.3 ^a^	34.0 *	2.3 *	81.7 ^b^	16.0	10.8 *	83.8 *^b^	5.4	<0.001
Vitamin C	0.0	100	0	0.0	100	0	0.5 *	99.5 *	0	0.000
Vitamin E	9.2 *	90.8 *^a^	0 *	53.8 *	46.2 *^b^	0	42.7 *	57.3 *^b^	0	<0.001

AR: average requirement; UL: tolerable upper intake level. The percentage for inadequacy was calculated by comparing intakes with EFSA recommendations (AR were used for zinc, vitamin A, and C; AI was used for selenium and vitamin E). Results are expressed in percentages (%). Usual Intake for two 24 h dietary recalls were used for zinc, selenium, and vitamin C and E. Average gram intake values from two 24 h dietary recalls were used for vitamin A. Chi-square test was used to evaluate differences by total and age group between REF and FMC groups (significant differences are marked with an asterisk * in the percentage values of FMC). Chi-square test analysis was used to calculate differences among age groups within subpopulations (significant differences are marked with superscript letters in the percentage value of subjects meeting the recommendations from each group). *p*-values for this test are included in the last column of the table. *p*-value < 0.05 was considered statistically significant.

**Table 4:** Relationship between sociodemographic characteristics and total antioxidant minerals and vitamin intakes of the two subpopulations.

	Reference Group (REF)				Fortified Milk Consumers (FMCs)			
	Total *n* = 687				Total *n* = 726			
(g/day)	Mean	β	CI (95%)	*p*	Mean	β	CI (95%)	*p*
**Zinc**	6.9294				8.4380			
Geographical area		−0.053	(−0.130)–0.024	0.003 *		0.021	(−0.078)–0.120	0.675
Family income		0.087	(−0.091)–0.266	0.001 *		−0.004	(−0.240)–0.232	0.973
Higher level of parents’ education		0.012	(−0.124)–0.147	0.000 *		−0.010	(−0.190)–0.169	0.908
**Selenium**	58.9901				51.5507			
Geographical area		−1.235	(−2.066)–(−0.404)	0.004 *		−0.496	(−1.245)–0.252	0.193
Family income		1.412	(−0.511)–3.334	0.003 *		1.428	(−0.357)–3.212	0.117
Higher level of parents’ education		0.147	(−1.315)–1.609	0.000 *		−0.786	(−2.142)–0.570	0.256
**Vitamin A**	625.8606				719.4928			
Geographical area		−6.088	(−23.872)–11.696	0.502		−0.607	(−14.012)–12.798	0.929
Family income		14.509	(−26.639)–55.656	0.489		3.467	(−28.493)–35.427	0.831
Higher level of parents’ education		36.387	5.099–67.675	0.023 *		−2.160	(−26.448)–22.127	0.861
**Vitamin C**	73.8804				107.0817			
Geographical area		2.086	0.786–3.387	0.002 *		2.577	1.210–3.944	0.000 *
Family income		0.420	(−2.589)–3.429	0.784		1.951	(−1.308)–5.210	0.240
Higher level of parents’ education		3.122	0.834–5.410	0.008 *		0.535	(−1.942)–3.012	0.672
**Vitamin E**	4.8900				8.6167			
Geographical area		−0.108	(−0.203)–(−0.013)	0.026 *		−0.078	(−0.183)–0.026	0.141
Family income		0.107	(−0.113)–0.327	0.342		−0.107	(−0.355)–0.142	0.399
Higher level of parents’ education		−0.079	(−0.246)–0.089	0.356		0.035	(−0.154)–0.223	0.719

Results are expressed as mean, beta standardized coefficient (β), confidence interval (CI) (95%), and *p*-values < 0.05 were considered statistically significant and are marked with an asterisk (*) symbol. Estimation of the parameters was achieved using covariance analysis.

## Supplementary Materials

The following supporting information can be downloaded at: https://www.mdpi.com/article/10.3390/nu14194132/s1, Table S1: Odds ratios and 95% confidence intervals for intake equal to or higher than the median of antioxidant minerals relative to family and personal factors in the reference group of the Nutritional Study in Spanish Pediatric Population (EsNuPI) (*n* = 707), Table S2: Odds ratios and 95% confidence intervals for intake equal to or higher than the median of antioxidant vitamins relative to family and personal factors in the reference group of the Nutritional Study in Spanish Pediatric Population (EsNuPI) (*n* = 707), Table S3: Odds ratios and 95% confidence intervals for intake equal to or higher than the median of antioxidant minerals relative to family and personal factors in Fortified Milk Consumers of the Nutritional Study in Spanish Pediatric Population (EsNuPI) (*n* = 741), Table S4: Odds ratios and 95% confidence intervals for intake equal to or higher than the median of antioxidant vitamins relative to family and personal factors in fortified milk consumers of the Nutritional Study in Spanish Pediatric Population (EsNuPI) (*n* = 741).
