# Supplementary material for: Dietary Intake, Nutritional Adequacy, and Food Sources of Selected Antioxidant Minerals and Vitamins; and Their Relationship with Personal and Family Factors in Spanish Children Aged 1 to <10 Years: Results from the EsNuPI Study
Source: Nutrients. 2022 Oct 5;14(19):4132. doi: 10.3390/nu14194132 (PMC9573671; doi:10.3390/nu14194132)
Supplement: Supplementary file 1 [file nutrients-14-04132-s001.zip › nutrients-1899665-supplementary.pdf]

Supplementary Table S1. Odds ratios and 95% confidence intervals for intake equal to or higher than the median of antioxidant minerals relative to family and personal factors in the reference group of the Nutritional Study in Spanish Pediatric Population (EsNuPI) (*n* = 707).

Reference Group (REF)							
		UI Zinc (mg/day)(≥P50)			UI Selenium (μg/day) (≥P50)		
**Factor**	**Subcategories**	**OR**	**CI**	***p***	**OR**	**CI**	***p***
Sex	Boys	1			1		
	Girls	1.26	0.90-1.78	0.184	1.55	1.09-2.21	0.014 *
Age ¥	1 to <3 years	1			1		
	3 to <6 years	0.08	0.04-0.13	0.000 *	0.05	0.03-0.09	0.000 *
	6 to <10 years	0.46	0.32-0.67	0.000 *	0.30	0.21-0.44	0.000 *
Number of feeding bottles or glasses of milk per day	Less than 2	1			1		
	2 or more	1.30	0.90-1.87	0.156	1.44	0.99-2.08	0.056
PAL	≥P50 by sex and age	1.15	0.81-1.63	0.423	1.09	0.76-1.56	0.640
Size of municipality (n)	50,000-300,000	1			1		
	>300,000	0.47	0.33-0.66	0.000 *	0.65	0.46-0.93	0.017 *
Family income (€)	≤1500	1			1		
	1501-2000	0.89	0.55-1.45	0.639	1.30	0.77-2.20	0.326
	≥2000	0.73	0.43-1.23	0.236	1.63	0.94-2.80	0.081
	Not known/no answer	1.34	0.85-2.09	0.205	1.51	0.95-2.40	0.084
Highest level of education achieved by one parent	≤10 years of education	1			1		
	Secondary education	0.70	0.42-1.17	0.177	0.55	0.34-0.89	0.016 *
	University studies	0.98	0.66-1.46	0.933	0.68	0.45-1.01	0.057
Anthropometry	z-height for age	0.85	0.47-1.55	0.595	1.09	0.59-2.00	0.791
	z-BMI for age	1.14	0.79-1.63	0.489	0.94	0.65-1.36	0.751

UI, Individual Usual Intakes; OR, odds ratio; CI, confidence intervals; PAL, physical activity level. z-BMI/age and z-height/age were defined according to World Health Organization international growth patterns. * $p \leq 0.05$ was considered statistically significant.

Supplementary Table S2. Odds ratios and 95% confidence intervals for intake equal to or higher than the median of antioxidant vitamins relative to family and personal factors in the reference group of the Nutritional Study in Spanish Pediatric Population (EsNuPI) (*n* = 707).

		Reference Group (REF)								
		Vit A (g/day) (≥P50)			UI Vit C (mg/day) (≥P50)			UI Vit E (mg/day)(≥P50)		
**Factor**	**Subcategories**	**OR**	**CI**	***p***	**OR**	**CI**	***p***	**OR**	**CI**	***p***
Sex	Boys	1			1			1		
	Girls	1.19	0.87-1.62	0.275	1.15	0.84-1.57	0.393	1.19	0.87-1.62	0.278
Age ¥	1 to <3 years	1			1			1		
	3 to <6 years	1.53	1.03-2.28	0.036 *	1.58	1.05-2.37	0.028 *	0.60	0.40-0.89	0.012 *
	6 to <10 years	1.33	0.94-1.89	0.108	0.83	0.58-1.19	0.311	1.02	0.72-1.44	0.924
Number of feeding bottles or glasses of milk per day	Less than 2	1			1			1		
	2 or more	0.92	0.66-1.28	0.614	0.81	0.58-1.14	0.228	1.07	0.76-1.49	0.714
PAL	≥P50 by sex and age	1.21	0.89-1.64	0.228	1.24	0.91-1.70	0.173	1.19	0.87-1.62	0.289
Size of municipality (n)	50,000-300,000	1			1			1		
	>300,000	0.92	0.68-1.26	0.617	0.74	0.54-1.02	0.063	0.78	0.57-1.07	0.127
Family income (€)	≤1500	1			1			1		
	1501-2000	0.85	0.83-1.34	0.478	0.75	0.47-1.20	0.233	0.77	0.49-1.21	0.250
	≥2000	1.09	0.67-1.75	0.735	0.94	0.58-1.56	0.798	1.31	0.81-2.11	0.264
	Not known/no answer	1.12	0.75-1.69	0.580	1.03	0.68-1.56	0.879	1.26	0.84-1.89	0.267
Highest level of education achieved by one parent	≤10 years of education	1			1			1		
	Secondary education	0.89	0.58-1.34	0.567	0.54	0.35-0.82	0.004 *	0.80	0.53-1.22	0.308
	University studies	0.70	0.50-1.00	0.050 *	0.54	0.38-0.78	0.001 *	0.68	0.48-0.97	0.031 *
Anthropometry	z-height for age	0.80	0.49-1.33	0.392	0.89	0.54-1.48	0.661	0.73	0.44-1.20	0.215
	z-BMI for age	0.88	0.82-1.51	0.654	0.99	0.71-1.38	0.934	1.09	0.78-1.52	0.607

UI, Individual Usual Intakes; OR, odds ratio; CI, confidence intervals; PAL, physical activity level. z-BMI/age and z-height/age were defined according to World Health Organization international growth patterns. * $p \leq 0.05$ was considered statistically significant.

Supplementary Table S3. Odds ratios and 95% confidence intervals for intake equal to or higher than the median of antioxidant minerals relative to family and personal factors in Fortified Milk Consumers of the Nutritional Study in Spanish Pediatric Population (EsNuPI) (*n* = 741).

Fortified Milk Consumers (FMC)							
		**UI Zinc (mg/day) (≥P50)**			**UI Selenium (µg/day) (≥P50)**		
**Factor**	**Subcategories**	**OR**	**CI**	***p***	**OR**	**CI**	***p***
Sex	Boys	1			1		
	Girls	0.96	0.68-1.35	0.823	0.79	0.55-1.13	0.198
Age ¥	1 to <3 years	1			1		
	3 to <6 years	0.06	0.04-0.10	0.000 *	0.04	0.02-0.06	0.000 *
	6 to <10 years	0.38	0.24-0.60	0.000 *	0.23	0.14-0.39	0.000 *
Number of feeding bottles or glasses of milk per day	Less than 2	1			1		
	2 or more	0.92	0.61-1.38	0.679	1.72	1.12-2.66	0.014 *
PAL	≥P50 by sex and age	1.23	0.88-1.74	0.229	1.47	1.02-2.12	0.041 *
Size of municipality (n)	50,000-300,000	1			1		
	>300,000	0.83	0.59-1.18	0.308	0.69	0.47-0.99	0.046 *
Family income (€)	≤1500	1			1		
	1501-2000	1.50	0.92-2.44	0.103	0.85	0.50-1.43	0.531
	≥2000	0.52	0.31-0.88	0.140	0.57	0.33-1.00	0.048 *
	Not known/no answer	1.01	0.66-1.56	0.955	1.29	0.81-2.06	0.277
Highest level of education achieved by one parent	≤10 years of education	1			1		
	Secondary education	0.94	0.56-1.58	0.814	1.17	0.67-2.04	0.573
	University studies	1.29	0.88-1.89	0.187	1.52	1.01-2.27	0.044 *
Anthropometry	z-height for age	1.08	0.66-1.76	0.762	2.25	1.33-3.79	0.002 *
	z-BMI for age	1.11	0.77-1.61	0.579	1.11	0.74-1.67	0.613

UI, Individual Usual Intakes; OR, odds ratio; CI, confidence intervals; PAL, physical activity level. The age group was used as the control variable in the analyses. z-BMI/age and z-height/age were defined according to World Health Organization international growth patterns. * $p \leq 0.05$ was considered statistically significant.

Supplementary Table S4. Odds ratios and 95% confidence intervals for intake equal to or higher than the median of antioxidant vitamins relative to family and personal factors in fortified milk consumers of the Nutritional Study in Spanish Pediatric Population (EsNuPI) (*n* = 741).

		Fortified Milk Consumers (FMC)								
		Vit A (g/day) (≥P50)			UI Vit C (mg/day) (≥P50)			UI Vit E (µg/day) (≥P50)		
**Factor**	**Subcategories**	**OR**	**CI**	***p***	**OR**	**CI**	***p***	**OR**	**CI**	***p***
Sex	Boys	1			1			1		
	Girls	0.94	0.70-1.27	0.698	1.06	0.79-1.43	0.695	0.48	0.20-1.15	0.099
Age ¥	1 to <3 years	1			1			1		
	3 to <6 years	1.62	1.10-2.38	0.015 *	1.22	0.83-1.81	0.313	0.84	0.27-2.61	0.759
	6 to <10 years	1.01	0.69-1.50	0.950	0.80	0.54-1.19	0.274	0.64	0.22-1.87	0.413
Number of feeding bottles or glasses of milk per day	Less than 2	1			1			1		
	2 or more	0.43	0.30-0.62	0.000 *	0.53	0.37-0.77	0.001 *	0.56	0.24-1.33	0.189
PAL	≥P50 by sex and age	0.43	0.30-0.62	0.000 *	0.92	0.68-1.25	0.590	0.73	0.31-1.69	0.462
Size of municipality (n)	50,000-300,000	1			1			1		
	>300,000	1.13	0.83-1.54	0.432	1.33	0.98-1.80	0.070	1.78	0.78-4.08	0.170
Family income (€)	≤1500	1			1			1		
	1501-2000	0.75	0.49-1.16	0.196	0.94	0.60-1.48	0.786	0.53	0.19-1.47	0.219
	≥2000	0.72	0.46-1.13	0.156	0.90	0.57-1.42	0.639	1.04	0.30-3.65	0.951
	Not known/no answer	1.01	0.68-1.49	0.962	1.02	0.69-1.51	0.920	1.84	0.53-6.42	0.340
Highest level of education achieved by one parent	≤10 years of education	1			1			1		
	Secondary education	0.86	0.54-1.35	0.504	0.63	0.41-0.97	0.037 *	0.49	0.15-1.60	0.235
	University studies	1.01	0.72-1.42	0.938	1.12	0.80-1.55	0.516	0.60	0.22-1.69	0.337
Anthropometry	z-height for age	0.86	0.56-1.32	0.485	0.61	0.40-0.93	0.022 *	0.90	0.28-2.89	0.861
	z-BMI for age	0.85	0.61-1.18	0.330	0.83	0.59-1.16	0.267	1.63	0.70-3.82	0.256

UI, Individual Usual Intakes; OR, odds ratio; CI, confidence intervals; PAL, physical activity level. z-BMI/age and z-height/age were defined according to World Health Organization international growth patterns. * $p \leq 0.05$ was considered statistically significant.
